# Roles of E3 ubiquitin ligases and deubiquitinating enzymes in renal cell carcinoma

**DOI:** 10.3389/fonc.2025.1628710

**Published:** 2026-01-21

**Authors:** Minshu Jiang, Wenxia Si, Sien Huang, Sha Qu, Minghui Zhang, Yi Quan

**Affiliations:** 1 Guangdong Medical University, Zhanjiang, Guangdong, China; 2 Department of Oncology Medical Center, The First People’s Hospital of Zhaoqing, Zhaoqing, Guangdong, China

**Keywords:** E3 ubiquitin ligases, deubiquitinating enzymes, ubiquitination, renal cell carcinoma, enzymes, targeted therapy

## Abstract

Ubiquitination is an important post-translational modification of proteins that precisely regulates protein stability and function through the coordinated actions of E3 ubiquitin ligases (E3s) and deubiquitinases (DUBs), participating in biological processes including protein degradation and signal transduction. In recent years, the role of ubiquitination modification in the carcinogenesis, progression, and treatment of renal cell carcinoma (RCC) has garnered increasing attention. This review summarizes the structural classifications of key enzymes in the ubiquitination process—E3s and DUBs—and to discuss their specific molecular mechanisms in RCC. Finally, we discuss the targeted therapeutic strategies focusing on these key ubiquitination-modifying enzymes in RCC.

## 1 Introduction

Renal cell carcinoma (RCC) is the most common malignant tumor of the adult kidney, accounting for 2.2% of all cancers globally. In 2022, the number of new cases of kidney cancer worldwide was approximately 400,000 (1). RCC is primarily divided into three subtypes: clear cell renal cell carcinoma (ccRCC), papillary renal cell carcinoma (pRCC), and chromophobe renal cell carcinoma (chRCC), among which ccRCC is the most common, representing 75% of renal cancers and being the main cause of renal cancer mortality (2). In China, RCC ranks second in the incidence of urogenital system tumors, following bladder cancer (3). In recent years, significant progress has been made in the treatment of RCC, evolving from traditional high-dose cytokine therapy combined with surgical tumor resection to targeted therapy, immunotherapy, and combination therapies (4). However, despite continuous advancements in treatment modalities, approximately 40% of RCC patients still experience tumor recurrence and metastasis, which ultimately lead to death (5). Therefore, exploring new therapeutic targets and developing novel anticancer drugs are crucial for improving the therapeutic outcomes of RCC patients.

Ubiquitination represents one of the core mechanisms of post-translational regulation, mediating the specific degradation of substrate proteins via the ubiquitin-proteasome system (UPS) and extensively participating in critical biological processes such as cell cycle regulation, DNA repair, and protein quality control.

**Figure 1:**
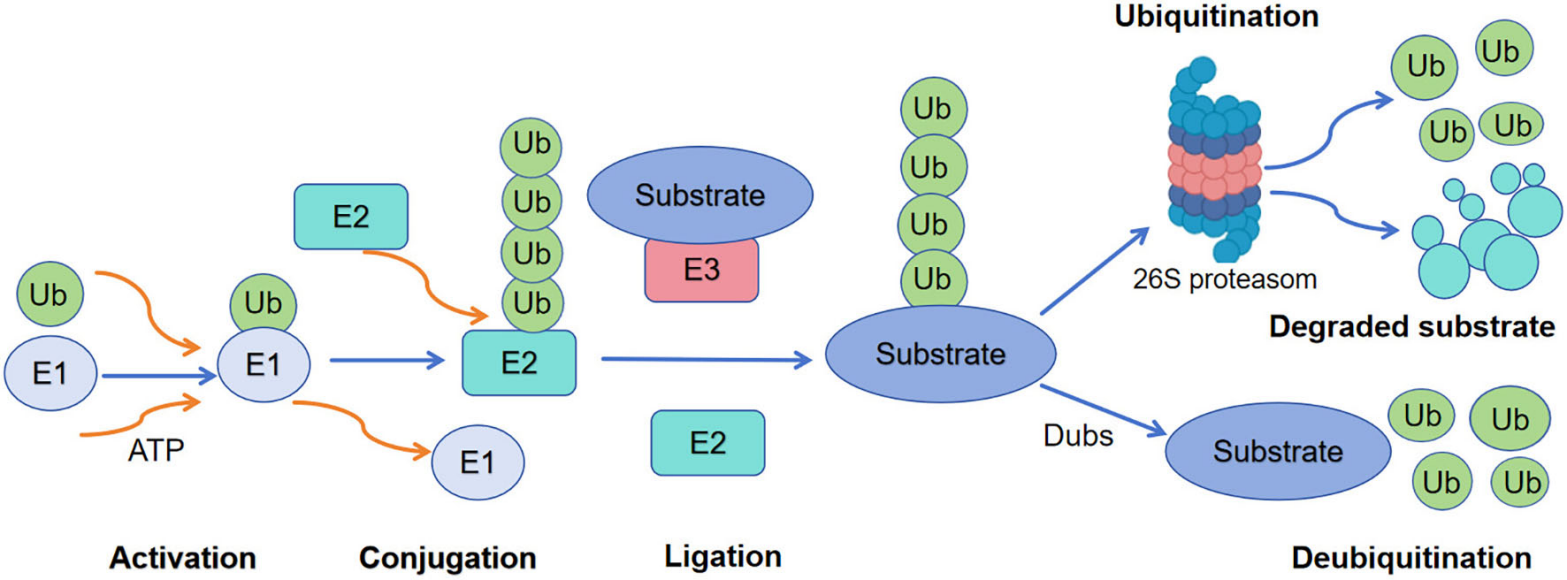
The processes of ubiquitination and deubiquitination Flowchart illustrating the ubiquitination process. It includes steps: Activation (E1 enzyme and ATP), Conjugation (E2 enzyme), and Ligation (E3 enzyme and substrate). Post-ubiquitination, the substrate is processed by the 26S proteasome into a degraded substrate and ubiquitin molecules are recycled. Deubiquitination involves Dubs and substrate regeneration.

The process of ubiquitination is regulated by ubiquitin-activating enzymes (E1), ubiquitin-conjugating enzymes (E2), ubiquitin ligases (E3), and deubiquitinating enzymes (DUBs) and involves three consecutive enzymatic reaction steps. First, ubiquitin-activating enzyme E1 hydrolyzes ATP and adenylylates a ubiquitin molecule, then forms a thioester bond with the C-terminal glycine of ubiquitin through its active center cysteine residue, thereby activating a single free ubiquitin. Subsequently, E1 transfers the activated ubiquitin to the cysteine residue of ubiquitin-conjugating enzyme E2. Finally, ubiquitin ligase E3 recruits specific substrates and E2, and mediates the transfer of ubiquitin from E2 to the target protein, resulting in the formation of an isopeptide bond between the ϵ-amino group of a specific lysine residue of the target protein and the C-terminal carboxyl group of the ubiquitin molecule, thus completing the ubiquitination modification (6–8). The ubiquitin molecule is attached to the target protein, forming a ubiquitination tag that directs the target protein into the proteasome for degradation (**Figure 1**) (9).

Flowchart illustrating the ubiquitination process. It includes steps: Activation (E1 enzyme and ATP), Conjugation (E2 enzyme), and Ligation (E3 enzyme and substrate). Post-ubiquitination, the substrate is processed by the 26S proteasome into a degraded substrate and ubiquitin molecules are recycled. Deubiquitination involves Dubs and substrate regeneration.

Moreover, ubiquitin chains exhibit remarkable diversity, being assembled through distinct lysine residues (K6, K11, K27, K29, K33, K48, K63) and a single methionine residue (Met1) (10), which determines their downstream signaling functions. The dynamic equilibrium of ubiquitination is stringently regulated by deubiquitinating enzymes, which catalyze the removal of ubiquitin chains and thereby reverse the effects of ubiquitination.

Ubiquitination plays a critical role in tumor cell survival, proliferation, and differentiation. Targeting E3 ubiquitin ligases and deubiquitinases enzymes has emerged as a key frontier in cancer research. Here, we systematically delineate the structural classification of E3 ubiquitin ligases and DUBs (**Table 1**) and their functional mechanisms in renal cancer (**Table 1**).

**Table 1:** Classification of E3 Ligase and Deubiquitinases.

Enzyme Category	Type	Subfamily/Family	Representative Members
E3s	HECT	NEDD4 family	Nedd4-1, Nedd4-2, ITCH, Smurfl-1, Smurfl-2, wwp1, wwp2, NEDL-1, NEDL-2
	HECT	HERC family	HERC1, HERC2
	HECT	Other HECT	E6AP, EDD, Huwel
	RING	Monomeric RING E3	MDM2, cIAP, TRAF6
	RING	Homo/Heterodimeric/RING E3	TRIM, MDM2, MDM2-MDMX, BRCA1-BARD1, RING1B/BMI-1
	RING	Multi-subunit RING E3	Cullin-RING Ligases(CRLs)
	RING	Other multi-subunit E3	APC/C Complex
	U-box	U-box E3	CHIP
	PHD	–	UBR7
DUBs	Cysteine Protease	UCH family	BAP1, UCHL5
	Cysteine Protease	USP family	USP2, USP7, UPS10, USP13, USP19, USP22, USP, 35, USP37, USP38, USP39, USP44, UPS46, USP53
	Cysteine Protease	OTU family	OTUD1, OTUDB1,OTUD6A, OTUD6B, OTD3
	Cysteine Protease	MJD family	JOSD1, JOSD2
	Cysteine Protease	ZUP1	ZUP1
	Metallproteinase	JAMM family	AMSH, BRCC3

## 2 Structure and classification of E3s

In the ubiquitination process, E3 ubiquitin ligases play a decisive role in recognizing and specifically tagging substrate proteins for ubiquitination. It is estimated that the human genome encodes over 600 types of E3s. Based on the specific domains involved in the transfer of ubiquitin to target proteins, E3s are classified into four categories (11, 12):(1) E3s containing the HECT (homologous to E6-AP carboxyl terminus) domain; (2) E3s containing the RING (really interesting new gene) domain; (3) E3s containing the U-box (Ubiquitin-protein ligases) domain; (4) PHD (plant homeodomain) finger domain (**Table 1**).

### 2.1 HECT domain-containing E3s

HECT E3 ligases represent the only class of enzymes capable of forming a thioester intermediate with ubiquitin and directly catalyzing target protein ubiquitination, with a domain architecture comprising an N-lobe (for substrate binding) and a C-lobe (accommodating ubiquitin transferred from E2 enzymes) (13, 14). Twenty-eight HECT E3 ubiquitin ligases have been identified in humans, which are classified into three distinct subfamilies based on their N-terminal domains: the NEDD4 family containing N-terminal WW and C2 domains (NEDD4-1, NEDD4-2, ITCH, SMURF1, SMURF2, WWP1, WWP2, NEDL1, NEDL2) (15), the HERC family harboring an N-terminal RCC1-like domain (RLD), and the HECT family with variable N-terminal domains (E6AP, EDD, HUWE1, etc.) (16). Among these, the NEDD4 family is the most numerous and functionally prominent.

### 2.2 RING domain-containing E3s

RING E3 ligases represent a class of prototypical ubiquitin ligases characterized by the RING domain, which harbors a conserved cysteine and histidine residue motif (Cys-X2-Cys-X(9-39)-Cys-X(1-3)-His-X(2-3)-Cys-X2-Cys-X(4-48)-Cys-X2-Cys) and coordinates two zinc ions to form a stable cross-braced structure that provides a binding platform for the E2-ubiquitin complex and directly catalyzes ubiquitin transfer onto substrate proteins (17, 18). Based on subunit composition and structural features, RING E3 ligases are categorized as follows.

1. Single-subunit RING E3 ligases function as monomers or oligomers, including monomeric forms such as MDM2, cIAP, and TRAF6, which independently mediate substrate recognition and ubiquitination; and homodimers and heterodimers such as MDM2-MDMX, BRCA1-BARD1, and RING1B-BMI1, which enhance stability and substrate selectivity through subunit cooperation (19–21).
2. Multi-subunit RING E3 complexes include Cullin-RING ligases (CRLs), the largest family of multi-subunit E3 ligases, comprising Cullin scaffold proteins, RING-box proteins (RBX1/2), adaptor proteins, and substrate recognition modules (22). Depending on the Cullin isoform (e.g., Cul1–Cul9), CRLs recognize distinct substrates; for example, CRL2VHL (CRL2 containing VHL) recognizes hypoxia-inducible factors via the VHL protein, playing a pivotal role in renal carcinogenesis (23).
3. Other complexes include the APC/C (Anaphase-Promoting Complex), which plays a central role in cell cycle regulation (24); and RING-between-RING (RBR) E3 ligases (e.g., Parkin and HOIP), which combine features of both RING and HECT ligases and form an E3-ubiquitin intermediate during catalysis, thereby expanding the mechanistic diversity of ubiquitination reactions (25).

### 2.3 U-box domain-containing E3s

The U-box is a domain containing approximately 70 amino acids, present in a variety of organisms from yeast to humans, that represents a critical E3 ligase domain in protein quality control pathways. Unlike RING domains, which rely on zinc ion coordination for structural stability, the U-box maintains its three-dimensional conformation through a specific hydrogen bond network and hydrophobic interactions without requiring metal ions. Despite these structural differences, U-box domains retain canonical E3 ligase activity, and missense mutations in key amino acids can result in complete loss of catalytic function (26).

Among this family, CHIP (C-terminus of HSC70-Interacting Protein) serves as a paradigmatic member that binds to molecular chaperones(including Hsp70/Hsp90) via its TPR domain, thereby utilizing the U-box domain to ubiquitinate misfolded or damaged proteins for proteasomal degradation (27). This mechanism establishes the central role of U-box E3 ligases in maintaining intracellular protein homeostasis, with particular significance in pathological processes associated with neurodegenerative diseases and cancer.

### 2.4 PHD domain-containing E3s

The PHD zinc finger is a domain featuring a characteristic Cys4-His-Cys3 motif that chelates two zinc ions to form a cross-braced topological conformation (28, 29). Recent studies have revealed that certain PHD-containing proteins exhibit non-canonical E3 ubiquitin ligase activity. Among these, UBR7 serves as a representative member whose PHD domain has been definitively demonstrated to possess independent E3 catalytic activity (30). Notably, UHRF1 and its homolog UHRF2 harbor both PHD and RING domains, where the RING domain mediates ubiquitination of substrates such as cyclin D1 and XRCC1 (31). Current research posits that although most PHD domains may primarily function as auxiliary substrate recognition modules, certain members (e.g., UBR7) indeed possess independent catalytic capacity, thereby offering a novel research direction for deepening our understanding of ubiquitination regulatory mechanisms (32).

## 3 The role of E3s in RCC

### 3.1 The role of HECT E3s in RCC

HECT E3 ligases exert critical regulatory functions in renal carcinogenesis by mediating the ubiquitination and degradation of tumor suppressors or oncogenic factors. Herein, we detail the specific roles of various HECT ligases in RCC.

#### 3.1.1 Smurf2

The NEDD4 family member Smurf2 has well-defined functions across multiple tumor types (33, 34), with particularly prominent roles in RCC. Smurf2 exhibits dual substrate specificity in RCC. Recent studies in ccRCC demonstrate that its high expression negatively correlates with TGF-β receptor II (TbR-II) protein levels, downregulating TGF-β signaling via ubiquitination-mediated degradation of TbR-II (35). In VHL-deficient models, CDK4/6 inhibitors stabilize Smurf2, enabling it to degrade HIF-1α (Hypoxia-Inducible Factor 1-alpha) under normoxic conditions, thereby establishing a VHL-independent negative feedback circuit. Clinically, high Smurf2 expression significantly correlates with prolonged disease-free survival (DFS) and overall survival (OS) in ccRCC patients (36). Collectively, Smurf2 modulates RCC progression through dual mechanisms targeting TbR-II and HIF-1α. However, the ubiquitin chain type preference for these two substrates and the precise details of their reversible regulation remain to be elucidated.

#### 3.1.2 NEDD4L

NEDD4 family E3 ligases are generally downregulated in renal carcinoma, with NEDD4L (NEDD4-like) showing the most significant reduction (37). NEDD4L expression is subject to multi-layered suppression in kidney cancer, with RNA-binding protein KSRP serving as the key regulator. In ccRCC, KSRP coordinately inhibits NEDD4L through transcriptional and post-transcriptional mechanisms: on one hand, it induces WT1-mediated transcriptional repression; on the other hand, it upregulates miR-629-5p and directly binds to AU-rich elements to promote mRNA degradation. This results in NEDD4L downregulation and aberrant activation of the PI3K/AKT pathway, ultimately driving tumor proliferation, metastasis, and epithelial-mesenchymal transition (38). In RCC, low NEDD4L expression compromises its ability to suppress proliferation and autophagy while promoting apoptosis through multi-substrate ubiquitination. In clear cell RCC (ccRCC), studies have demonstrated that low NEDD4L expression inversely correlates with RAC2 (Rac family small GTPase 2) levels and associates with shortened overall survival and disease-specific survival. Mechanistically, NEDD4L binds to the Thr108-Pro motif of RAC2 and catalyzes K48-linked ubiquitination and degradation, thereby inhibiting proliferation and inducing apoptosis (39). Additionally, research has reported low NEDD4L expression in ccRCC, where it mediates ubiquitination and degradation of ERBB3 (Erythroblastic Leukemia Viral Oncogene Homolog 3), consequently suppressing MAPK signaling (40) NEDD4L has also been implicated in regulating ULK1 degradation during autophagy (41, 42). Under nutrient deprivation, NEDD4L promotes ULK1 ubiquitination and degradation to prevent excessive autophagy; conversely, NEDD4L knockdown enhances autophagy. Together, NEDD4L’s “low expression–multi-substrate” pattern offers a novel prognostic biomarker panel for ccRCC.

#### 3.1.3 HERC

HERC1 and HERC2, as HECT family E3 ubiquitin ligases, influence cell proliferation by regulating signaling pathways. For instance, HERC1 catalyzes K48-linked polyubiquitination of C-RAF (RAF Proto-Oncogene Serine/Threonine-Protein Kinase), promoting its degradation; HERC1 knockdown stabilizes C-RAF, increases ERK phosphorylation, and accelerates cell proliferation. *In vitro* ubiquitination assays further validate C-RAF as a HERC1 substrate, indicating that HERC1 negatively regulates ERK signaling by controlling C-RAF stability (43). Similarly, HERC2 ubiquitinates C-RAF to regulate its protein levels and mediates cellular oxidative stress responses, thereby influencing antioxidant gene expression through modulation of the C-RAF/MKK3/P38 signaling axis. Clinically, HERC1/2 expression levels positively correlate with survival rates in RCC patients (44), underscoring their prognostic value. Notably, although HERC1 and HERC2 share C-RAF as a common substrate, they predominantly govern proliferative and stress signaling pathways, respectively; the mechanistic basis for their spatiotemporal functional divergence warrants further investigation.

### 3.2 The role of RING E3s in RCC

Accumulating evidence demonstrates that RING family E3 ubiquitin ligases exert pivotal functions in renal carcinogenesis by orchestrating the ubiquitination and degradation of oncogenic or tumor-suppressive factors.

#### 3.2.1 FBXW7

FBXW7 is frequently inactivated in RCC through mechanisms encompassing genetic alterations, transcriptional repression, and aberrant protein stability/complex assembly. Specifically: the FBXW7 gene can be disrupted by chromosomal translocation t(3;4)(q21;q31) in certain RCC cases, with concomitant loss of the derivative chromosome resulting in functional deletion (45); approximately 4% of Wilms tumors harbor FBXW7 mutations or deletions, and 8.7% (9/104) of cases exhibit amplification of MYCN—MLST8 (mTOR substrate for FBXW7, MLST8-mediated ubiquitination and degradation) (46); Functional miRNAs such as miR-92a-3p are significantly overexpressed in RCC, inhibiting FBXW7 translation by targeting its 3’ UTR and serving as key upstream regulators driving FBXW7 downregulation (47). Clinical sample analyses reveal that FBXW7 expression in RCC tissues is significantly lower than in adjacent normal tissues and negatively correlates with pathological grade and TNM stage (48). Functionally, FBXW7 serves as the substrate recognition component of the SCF (SKP1-CUL1-F-box protein) E3 ubiquitin ligase complex, exerting tumor-suppressive activity through K48-linked polyubiquitination and proteasomal degradation of oncogenic drivers such as cyclin E, c-Myc, c-Jun, Notch-1, and AURKA (49); notably, deletion of its F-box domain abrogates assembly of the functional SCF complex (50) Collectively, FBXW7 inactivation drives RCC progression through multi-level mechanisms initiated by genetic alterations, accelerated transcriptional silencing by miRNAs, decreased protein stability, and functional complex disassembly. Specifically, FBXW7 inactivation precipitates dysregulation of three major oncogenic axes. For instance, FBXW7 inactivation promotes clear cell RCC (ccRCC) progression through MLST8(MTOR-associated protein,MLST8 homolog)-driven oncogenic signaling. Mechanistically, CDK1 phosphorylates FBXW7 to enable recognition of the (T/S)PXX(S/T/D/E) degron motif in MLST8 and subsequent K48-linked ubiquitination and degradation. FBXW7 loss or CDK1 overexpression leads to MLST8 accumulation, mTOR pathway activation, and enhanced cell proliferation and migration. *In vitro* studies confirm that this axis serves as a critical mediator of FBXW7 inactivation-induced tumorigenesis (51).

FBXW7 orchestrates a positive feedback loop with its substrates NFAT1 (Nuclear Factor of Activated T Cells 1) and PRR11 (Proline-rich Protein 11) to drive ccRCC progression. NFAT1, an FBXW7 substrate involved in tumor immunity, is aberrantly overexpressed in ccRCC and associated with poor prognosis. It is stabilized via hyperactivation of the PI3K/AKT/GSK-3β axis, leading to PD-L1 upregulation, immune escape, and resistance to sunitinib. Moreover, FOXA1/SETD2 downregulation causes FBXW7 loss, allowing NFAT1 to evade FBXW7-mediated K48 ubiquitination and degradation, thereby establishing a positive feedback circuit (52). PRR11 represents another FBXW7 substrate that undergoes GSK3β phosphorylation followed by FBXW7-catalyzed K48 ubiquitination and degradation. However, PRR11 concurrently activates AKT, which inhibits GSK3β activity, enabling PRR11 to escape degradation, block DNA oxidative damage, and accelerate RCC progression (53).

The FBXW7 substrate repertoire in renal cancer exhibits a “functional trifurcation” pattern: MLST8, NFAT1, and PRR11 correspond to three core events—metabolic reprogramming, immune evasion, and genomic stability—establishing FBXW7 as a molecular hub. Current studies are limited to single-substrate validation; construction of a co-degradation atlas to delineate pathway crosstalk and assessment of therapeutic windows and toxicities for combinatorial interventions are urgently needed. In RCC, FBXW7 inactivation results from the synergistic cooperation of genetic events and aberrant signaling pathways, wherein functional sequestration of FBXW7 due to AKT-mediated GSK3β inactivation is an important RCC-specific mechanism that provides a novel conceptual framework for for targeted therapy.

#### 3.2.2 MDM

MDM2 (491 amino acids) exerts E3 ligase activity via its C-terminal RING finger domain to catalyze p53 ubiquitination and degradation, thereby antagonizing p53’s tumor-suppressive function (54). MDM2 dysregulation in kidney cancer stems from multi-layered regulatory abnormalities, with its overexpression driven by the combined effects of gene amplification, transcriptional activation, and pathway crosstalk. For instance, in a subset of ccRCC, amplification of the 12q14–15 chromosomal region where MDM2 resides directly increases gene copy number (55). Furthermore, sustained activation of the p53-MDM2 negative feedback loop also contributes to this process (56). These abnormalities collectively lead to MDM2 overexpression; clinical data demonstrate that, in clear cell RCC (ccRCC), MDM2 positivity reaches 54.5%, and MDM2/p53 co-expression correlates with shortened overall survival (OS) (57), suggesting that targeting the MDM2-p53 axis represents a strategy for restoring p53 function and inhibiting RCC progression.

#### 3.2.3 TRIMs

TRIMs (Tripartite Motif) proteins represent a family of RING finger domain-containing E3 ubiquitin ligases. Certain members exhibit aberrant expression in malignancies, driving tumorigenesis and progression by orchestrating dysregulated signaling pathways through substrate ubiquitination (58). In recent years, an increasing number of studies have shown that members of the TRIM members are also involved in the regulation of RCC. Specifically, TRIM21, TRIM44, TRIM65 and TRIM47 have emerged as key regulatory factors in this area, which is attributed to their well-elucidated mechanisms and clinical relevance in tumor initiation and progression.

TRIM21 as a multifunctional tumor suppressor. Studies have revealed that TRIM21 is downregulated in primary ccRCC and predicts poor prognosis. TRIM21 suppresses tumor cell glycolysis, proliferation, tumorigenesis, migration, and metastasis by ubiquitinating and degrading HIF-1α (59). Additionally, TRIM21 negatively regulates lipogenesis by mediating K48-linked ubiquitination and degradation of SREBF1 (Sterol Regulatory Element-Binding Transcription Factor 1), which downregulates fatty acid synthase and reduces intracellular lipid content (60). Regarding tumor microenvironment regulation, AXL (AXL Receptor Tyrosine Kinase) is an independent prognostic indicator of poor outcome in KIRC; its stability is subject to competing regulation by TRIM21 and the deubiquitinase STAMBPL1 (STAM Binding Protein-Like 1). TRIM21 promotes AXL degradation via K48-linked ubiquitination, thereby suppressing EMT and stem cell phenotypes, while enhancing CD8^+^ T cell function to improve immunotherapy sensitivity (61). ASS1 (Argininosuccinate Synthetase 1), a key enzyme in the urea cycle whose aberrant expression is linked to tumor progression in multiple cancers (62), is stabilized by TRIM21 through K63-linked ubiquitination, leading to suppression of the mTORC1-EMT axis and inhibition of ccRCC cell invasion and migration (63). This metabolic–metastatic pathway represents a recent discovery, and functional characterization remains limited to ccRCC models. Collectively, TRIM21 integrates hypoxia metabolism, lipogenesis, receptor signaling, and the immune microenvironment, constituting a reversible tumor-suppressive hub in ccRCC.

TRIM44 also as a tumor suppressor; TRIM44 functions as an E3 ubiquitin ligase in RCC (64). TRIM44 is upregulated in RCC tissues and mediates FRK (Fyn-Related Kinase) ubiquitination and degradation (65). TRIM44 also degrades the EMT core marker vimentin via K48-linked ubiquitination, thereby inhibiting RCC invasion and migration (66).

Furthermore, TRIM47 and TRIM65 have been identified as oncogenic factors in RCC, both of which are highly expressed and associated with shortened overall survival. TRIM47 drives tumor progression by enhancing its E3 ubiquitin ligase activity to promote p53 ubiquitination and degradation, thereby abrogating p53’s tumor-suppressive function (67); TRIM65 catalyzes K48-linked ubiquitination and degradation of BTG3 (BTG anti-proliferation factor 3) at lysine 41, alleviating G2/M phase arrest and promoting proliferation. Clinical tissue microarray analysis reveals that TRIM65 expression negatively correlates with BTG3 levels (68).

In summary, TRIM family E3 ubiquitin ligases display a bidirectional regulatory pattern in RCC, with certain members downregulated (e.g., TRIM21) and others upregulated (TRIM44, TRIM47, TRIM65), and their expression levels are significantly correlated with prognosis. Among them, TRIM21 exhibits the most pleiotropic functions, acting as a critical tumor suppressor hub by orchestrating hypoxic metabolism, lipid synthesis, receptor signaling, and the immune microenvironment through mechanisms including ubiquitin-mediated degradation of HIF-1α, SREBF1, and AXL, as well as stabilization of ASS1. TRIM47 and TRIM65 exert oncogenic roles through degrading p53 and BTG3, respectively. Collectively, TRIM proteins regulate multiple substrates to participate in fundamental processes of ccRCC, including proliferation, metabolism, EMT, and immune evasion. Their expression profiles and functional heterogeneity provide potential biomarkers for prognostic assessment and therapeutic windows for targeted intervention.

Nevertheless, limitations persist. Notably, the upstream mechanisms—such as gene amplification, transcriptional activation, and signaling pathway regulation—underlying the upregulated expression or enhanced activity of certain TRIM proteins in RCC remain poorly defined. This constitutes a critical knowledge gap in the field and a limitation that must be explicitly acknowledged.

#### 3.2.4 VHL

**Figure 2:**
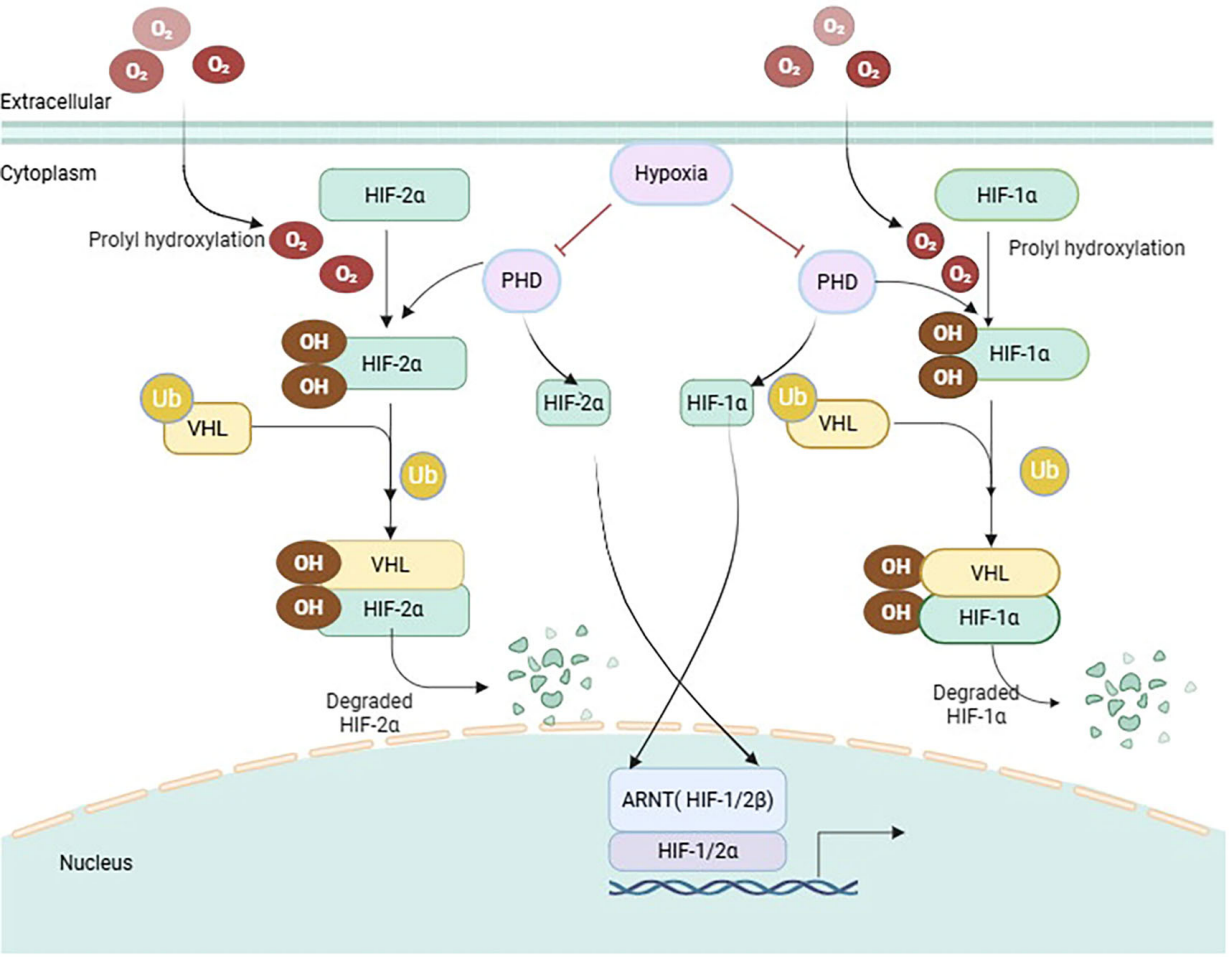
Hypoxia and Oxygen Sensing in ccRCCIllustration of the cellular response to oxygen levels, showing the pathways of HIF-1a and HIF-2a under normoxia and hypoxia. Under normoxia, prolyl hydroxylation leads to VHL-mediated degradation of HIF proteins. Hypoxia inhibits prolyl hydroxylation, stabilizing HIFs, which translocate to the nucleus to form complexes with ARNT, affecting gene expression.

VHL (Von Hippel-Lindau) belongs to the Cullin-2 family of E3 ubiquitin ligases. In ccRCC, the VHL protein functions as the substrate recognition subunit of the Cullin-2 RING-E3 ligase complex, coordinating with elongin B/C, Cullin-2, and Rbx1 to mediate substrate ubiquitination and proteasomal degradation (69, 70). Specifically, hypoxia and oxygen-sensing mechanisms in ccRCC center on the “VHL-PHD-HIF” axis, accompanied by multiple bypass pathways and metabolic reprogramming. Under normoxic conditions, PHD1/2/3 hydroxylate HIF-α at Pro402/564 in the presence of O_2_, Fe²^+^, and 2-oxoglutarate, then CRL2VHL recognizes this hydroxylated degron and mediates K48-linked ubiquitin chain assembly, targeting HIF-α for rapid degradation by the 26S proteasome; conversely, under hypoxia or in the presence of Fe²^+^/CoCl_2_ mimetics, hydroxylation is impeded, allowing HIF-α to escape degradation and activate downstream oncogenic transcriptional programs (71–75) (**Figure 2**). Notably, loss of the short arm of chromosome 3 (3p) occurs in over 90% of sporadic ccRCC, leading to single-copy loss of the VHL gene. This 3p deletion concurrently causes haploinsufficiency of adjacent tumor suppressor genes (PBRM1, BAP1, SETD2), forming a cooperative oncogenic network with VHL (76). Aberrant hypermethylation of the CpG island in the 5’ region of the VHL gene directly inhibits gene transcription, resulting in loss of protein expression and producing effects equivalent to a gene mutation (77). Approximately 20% of cases achieve biallelic inactivation of VHL through promoter methylation without accompanying gene sequence alterations (78). In summary, VHL inactivation can be achieved through genetic (point mutations, deletions) and/or epigenetic (promoter methylation) mechanisms, constituting the earliest driver event. VHL targets hydroxylated HIF-1/2α for ubiquitination and degradation. Inactivating mutations in VHL can lead to the accumulation of HIF-1/2α, thereby causing the HIF signaling pathway to be abnormally activated (79). Moreover, HIF-1α predominantly drives angiogenesis and proliferation, whereas HIF-2α promotes invasion and inflammatory responses (80). Beyond the aforementioned classical substrates, CRL2VHL catalyzes the ubiquitin-proteasomal degradation of additional substrates, including ZHX2, SFMBT1, NDRG3, UBE3B, and AURKA. Specifically, ZHX2 undergoes hydroxylation at Pro427/440/464 and is subsequently recognized by VHL, thereby facilitating ccRCC progression through regulating NF-κB/p65 nuclear localization (81–84). At the metabolic level, studies have shown that in ccRCC, the high expression of HIF-1α mediated by VHL inactivating mutations promotes lipid synthesis by upregulating ACLY (acetyl-CoA carboxylase), thereby facilitating tumor progression (85). Additionally, it has been found that the absence of VHL in sporadic ccRCC specimens is associated with a significant increase in autophagy levels, and this elevated autophagy is related to poor patient prognosis. In the autophagy axis, further research has revealed that VHL inhibits autophagy through a PHD1-dependent hydroxylation mechanism of Beclin1, thereby suppressing tumor growth; this indicates that the loss of VHL not only promotes autophagy but is also associated with tumor growth and poor prognosis. Conversely, VHL deficiency leads to hyperactivation of autophagy, which correlates with poor prognosis (86). Moreover, at the epitranscriptomic level, studies have found that VHL regulates the formation of the METTL3/METTL14 complex (the main components of the m6A methyltransferase complex), thereby affecting the stability and protein levels of PIK3R3 mRNA, and consequently modulating the PI3K/AKT signaling pathway, which ultimately impacts the occurrence of renal tumorigenesis (87). Meanwhile, the function of VHL in renal cancer is also closely related to the immune microenvironment. Specifically, VHL mutations reduce naive CD4^+^ T cell infiltration and increase the proportion of memory CD4^+^ T cells (88). Moreover, VHL regulates the expression of VCAM-1 in an NF-κB-dependent manner, thereby promoting the immune response against RCC (89). In summary, the mechanisms of action of VHL in ccRCC are complex and diverse, involving multiple aspects, including the HIF, PI3K/AKT, and lipid metabolism pathways, and the immune microenvironment. Further research into the functions and related mechanisms of VHL will provide new ideas and strategies for the precise diagnosis and treatment of ccRCC.

Illustration of the cellular response to oxygen levels, showing the pathways of HIF-1a and HIF-2a under normoxia and hypoxia. Under normoxia, prolyl hydroxylation leads to VHL-mediated degradation of HIF proteins. Hypoxia inhibits prolyl hydroxylation, stabilizing HIFs, which translocate to the nucleus to form complexes with ARNT, affecting gene expression.

### 3.3 The role of U-box E3s in RCC

Studies have found that CHIP is downregulated in RCC and correlates with TNM staging and poor prognosis (90). It inhibits the proliferation of renal cancer cells by suppressing the phosphorylation of AKT and upregulating the expression of p21 (91). Future investigations could explore the detailed mechanisms through which CHIP regulates the AKT/p21 signaling axis.

### 3.4 The role of PHD E3s in RCC

Research on the PHD family in RCC remains limited. Currently, UHRF1 has been found to be upregulated in RCC and correlates with tumor stage, grade, poor prognosis, immune infiltration, aberrant DNA methylation, and mutation burden. Functionally, siRNA-mediated UHRF1 knockdown significantly inhibits RCC proliferation and induces apoptosis (92). In contrast to UHRF1’s oncogenic role, UBR7 is commonly downregulated in clear cell renal cell carcinoma(ccRCC) and papillary RCC (pRCC), and its low expression is associated with shortened overall survival (93). UBR7 exerts PHD domain-dependent E3 ligase activity (30); however, its substrate spectrum and preferred ubiquitin chain types remain undefined. The differential expression of these proteins confers distinct roles in RCC, warranting further investigation into their specific molecular mechanisms and the broader exploration of PHD family members.

## 4 Structure and classification of DUBs

DUBs antagonize proteasomal or lysosomal degradation by specifically removing substrate ubiquitin chains, thereby regulating protein stability and function and participating in critical biological processes such as cell cycle, apoptosis, and DNA repair. To date, more than a hundred DUBs have been identified in humans. Based on their structural and functional characteristics, DUBs are mainly classified into the following seven families (**Table 1**): (1) Ubiquitin C-terminal hydrolases (UCH) family; (2) Ubiquitin-specific proteases (USP) family; (3) OTU (ovarian tumor) protease family; (4) MJD (Josephin domain) family; (5) JAMM family containing the MPN+ (Metalloprotease of the N-terminus) domain; (6) Zinc-finger ubiquitin peptidase 1 (ZUP1); (7) Motif interacting with ubiquitin-containing DUBs (MINDY) (94, 95). The USP subfamily (56 members) constitutes the largest group, followed by the OTU (17), JAMM (12), MJD (4), UCH (4), MINDY (5), and ZUP1 (1) families. Except for the JAMM family, which is a zinc-dependent metalloprotease, the rest are cysteine proteases. Among these, 11 pseudoenzyme members, concentrated in the JAMM family, lack catalytic activity but still exert non-enzymatic functions. The MINDY and ZUP1 subfamilies were identified more recently. MINDY members specifically recognize K48-linked ubiquitin chains, thereby regulating protein homeostasis (96). ZUP1, a single-member family, binds K63-linked chains via a helical arm and UBZ domain to maintain genome stability and promote DNA damage repair (97).

### 4.1 The role of DUBs in RCC

Deubiquitinating enzymes (DUBs) play a critical regulatory role in RCC by antagonizing E3 ubiquitin ligases to stabilize key oncoproteins or tumor suppressor proteins. The USP family, representing the largest DUB subfamily, features a catalytic domain composed of three conserved subdomains and encompasses multiple members, several of which have been identified as pivotal factors driving or inhibiting tumor progression (98).

#### 4.1.1 The role of USPs in RCC

Some USPs act as carcinogenic factors, such as USP7, which is one of the most functionally versatile oncogenic DUBs. USP7 promotes RCC cell proliferation and G1/S phase progression by deubiquitinating and stabilizing ARMC5 (Armadillo Repeat Containing 5) (99). Intriguingly, USP7 overexpression in RCC predicts poor prognosis. USP7 inhibition blocks tumor progression *in vitro* and *in vivo*, as FUBP1/3 transcriptionally activates USP7 to subsequently stabilize HIF-2α, forming an oncogenic axis. Afatinib synergistically induces cell death by inhibiting FUBPs and augmenting USP7 downregulation-mediated HIF-2α degradation (100). USP13, identified as a ZHX2 deubiquitinase through DUB cDNA library screening, stabilizes ZHX2 protein via deubiquitination and modulates the ZHX2-NF-κB target gene signature. Knockdown of USP13 significantly inhibits ccRCC colony formation and anchorage-independent growth (101).

USP35 is another important oncogene. USP35 is highly expressed and independently predicts poor survival. It deubiquitinates the IAP family and NRF2 to suppress apoptosis and ferroptosis; knockdown downregulates NRF2 antioxidant target genes and enhances ferroptosis sensitivity. Additionally, USP35 silencing significantly reduces ccRCC xenograft tumor formation (102). Metabolomic analyses further reveal that high USP35 expression activates glycerophospholipid/linoleic acid oncogenic pathways, whereas its downregulation shifts metabolism toward nitrogen/purine metabolism and enhances immunogenicity (103), revealing its novel function in metabolic reprogramming.

Although the substrates of USP39 associated with its canonical DUB activity remain elusive, studies have demonstrated that knockdown of USP39 significantly inhibits AKT phosphorylation at Ser473, blocks AKT signaling activation, and thereby suppresses RCC metastasis and migration (104). In addition, USP39 was found to be highly expressed in RCC and associated with poor survival. USP39 binds SRPK1 (Serine/arginine-rich splicing factor kinase 1) through its 101–565 fragment, promoting SRPK1-SRSF1 (Serine and arginine-rich splicing factor 1) phosphorylation and interaction, which subsequently regulates VEGF-A165b alternative splicing to drive RCC proliferation and angiogenesis (105). Additionally, USP22 is co-upregulated with survivin (an apoptosis inhibitor) in RCC and correlates with poor prognosis; it deubiquitinates and stabilizes survivin, blocks apoptosis, and drives proliferation (106). USP37 binds to HIF-2α and promotes its deubiquitination. Functionally, USP37 knockdown leads to HIF-2α downregulation, inhibition of MTS/2D/3D proliferation, and a significant reduction in orthotopic renal tumorigenesis and spontaneous lung metastasis (107).

Some USPs function as tumor suppressor; for example, USP10 is significantly downregulated in ccRCC, leading to p53 destabilization and loss of growth suppression. Mechanistically, it catalyzes p53 deubiquitination, antagonizes MDM2, and promotes p53 nuclear re-entry. Upon DNA damage, ATM phosphorylates USP10, promoting its nuclear translocation and enhancing p53 stability (108). USP44 is downregulated in ccRCC and correlates with advanced stage, high grade, and poor prognosis. It suppresses proliferation and migration by deubiquitinating and stabilizing p21, degrading Cyclin D1, and negatively regulating JNK phosphorylation (109). USP53 is downregulated in ccRCC and correlates with tumor stage and poor prognosis; its downregulation promotes cell proliferation *in vitro* and *in vivo*. Additionally, high levels of USP53 increase the expression of IκB (Inhibitor of κB) and demonstrated that USP53 inhibits the activation of the NF-κB (Nuclear Factor kappa B) pathway by reducing the ubiquitination of IκB, thereby suppressing the proliferation and metastasis of ccRCC cells (110).

Collectively, in ccRCC/RCC, USP7, USP35, USP39, USP22, and USP37 are highly expressed, driving proliferation, ferroptosis evasion, and metabolic reprogramming by stabilizing NRF2, HIF-2α, AKT, and survivin. Validated inhibitors or knockdown rapidly suppress tumor growth. In contrast, USP10, USP44 and USP53 are generally downregulated, resulting in loss of deubiquitinase-mediated restraint on key pathways including NF-κB, JNK and p53. Re-expression or downstream pathway inhibitors can restore tumor-suppressive functions. Differential expression independently predicts poor prognosis. Future studies should elucidate specific molecular mechanisms, such as dissecting FUBPs-USP7 transcriptional regulatory details to optimize inhibitor design. However, USP13 has only been reported once as a ZHX2 stabilizer, and its substrate repertoire and chain topology (K48/K63) remain unexpanded. USP39 also exhibits dual functions in AKT signaling and VEGF splicing, requiring validation of which serves as the primary oncogenic driver.

#### 4.1.2 The role of OTUs in RCC

In addition to the USP family, members of the OTU family play pivotal roles in RCC by regulating core biological processes such as tumor signaling, immune response, and cell death, thereby influencing disease progression.

Members of the OTU family exert diverse regulatory functions in RCC by stabilizing specific substrates. Among them, OTUD1 has been confirmed as an important tumor suppressor. OTUD1 is downregulated in RCC and predicts poor prognosis, exerting tumor-suppressive functions by deubiquitinating and stabilizing PTEN (Phosphatase and Tensin homolog) to regulate the PI3K/AKT and TNF-α/NF-κB signaling pathways (111). Another study revealed that OTUD1 also deubiquitinates and stabilizes STAT3 (Signal Transducer and Activator of Transcription), blocking its nuclear translocation and PD-L1 transcription to enhance anti-tumor immunity (112). This forms a unique anti-cancer immune microenvironment.

In contrast, OTUB1 acts as a clear oncogene. OTUB1 is upregulated in RCC and correlates with poor prognosis. Further studies reveal that OTUB1 stabilizes FOXM1 (Forkhead box protein M1) through deubiquitination, upregulates epithelial cell transforming sequence 2 (ECT2) to activate Rho signaling, and promotes proliferation and migration *in vitro*; FOXM1 re-expression rescues these phenotypes (113). Additionally, OTUB1 is highly expressed in RCC and positively correlates with PD-L1 abundance; it blocks ERAD-mediated degradation of PD-L1 by deubiquitinating K48-linked chains, thereby enhancing PD-1 binding and suppressing CD8^+^ T cell infiltration and IFN-γ secretion (114).

In terms of metabolism and stress regulation, OTUD3 is highly expressed in ccRCC and promotes cell proliferation, suppresses ferroptosis, and affects cellular reactive oxygen species (ROS) levels by deubiquitinating and stabilizing SLC7A11 (Solute carrier family 7 member 11) (115). In RCC tissues, both OTUD6A and CDC6 (Cell Division Cycle 6) protein levels are elevated. OTUD6A promotes tumor proliferation and chemoresistance by deubiquitinating CDC6 and cleaving K6/K33/K48-linked ubiquitin chains, thereby antagonizing proteasomal degradation, activating the ATR-Chk1 signaling pathway, and enhancing DNA repair (116). OTUD6B is also an oncogene (117). In ccRCC patients with VHL missense mutations, low OTUD6B expression predicts shorter overall survival. OTUD6B knockdown enhances ccRCC cell migration and HIF-2α levels in a mutant VHL protein-dependent manner, whereas OTUD6B overexpression reverses these phenotypes (118).

#### 4.1.3 The role of UCHs in RCC

BAP1 (BRCA1-associated protein 1) belongs to the UCH subfamily of deubiquitinating enzymes (DUBs) (119). BAP1 is a key inactivated gene in RCC (120), whose inactivation is primarily achieved through a “two-hit” model involving chromosome 3p deletion and gene mutation. This process synergizes with VHL inactivation to drive the development and progression of high-grade, metabolically reprogrammed, and immune-infiltrated ccRCC. Following BAP1 inactivation, its diverse functions as a deubiquitinase are compromised (76). Studies have identified BAP1 inactivation in RCC and demonstrated its association with poor prognosis. It suppresses proliferation by binding HCF-1 (Host Cell Factor-1) to recruit chromatin-remodeling complexes; loss of this function leads to cell cycle dysregulation (121). Another member, UCHL5, plays a cancer-promoting role. UCHL5 is highly expressed in the UCH family and correlates with poor prognosis. It drives EMT and metastasis by deubiquitinating and stabilizing Snail1 (Snail family transcriptional repressor 1), and is associated with impaired antigen presentation by tumor-infiltrating B cells (122).

OTU/UCH deubiquitinases exert dual roles in RCC. Tumor-suppressive members (OTUD1, BAP1) inhibit tumor growth and enhance immunity by stabilizing substrates such as PTEN, STAT3, among others. Oncogenic members (OTUB1, OTUD6A/6B, OTUD3, UCHL5) activate proliferation, immune evasion, DNA repair, and EMT pathways by stabilizing substrates, including FOXM1, PD-L1, CDC6, SLC7A11, and Snail1, leading to poor prognosis. Differential expression among members collectively forms a deubiquitination regulatory network in RCC (**Table 2**).

**Table 2:** Roles of E3s and DUBs in RCC.

Category	Name	Expression Change	Substrates/Functional Node	Involved signal/Pathway	Driver event in RCC	Ref.
Oncogenic E3 Ligases	MDM2	↑	p53	p53	Ubiquitin-mediated degradation of p53 → loss of growth suppression	(54)
	Smurf2	↑	TbR-II HIF-1α	TGF-β	Dual degradation → dampens TGF-β & hypoxia pathways, linked to longer DFS/OS	(35, 36)
	TRIM47	↑	p53	P53	Ubiquitin-dependent degradation of p53 → loss of genomic guardian function	(67)
	TRIM65	↑	BTG3 (K48)	–	BTG3 destabilization → G2/M checkpoint failure → cell cycle acceleration	(68)
	UHRF1	↑	–	–	Pro-proliferation/anti-apoptosis axis ↑	(92)
Tumor Suppressor E3 Ligases	VHL	inactivation	HIF-1α HIF-2α ZHX2	HIF-1/2α, NF-κB	VHL inactivation → HIF-1/2α stabilization → activation of hypoxic response, angiogenesis, metabolic reprogramming, and sustained autophagy; NF-κB-mediated VCAM-1 expression to promote anti-tumor immunity	(80, 85, 86, 88, 89)
	FBXW7	↓	MLST8 NFAT1 PRR11	mTOR	Loss of degradation of three axes: mTOR metabolism ↑, PD-L1 immune evasion ↑, AKT-genomic instability ↑	(51–53)
	NEDD4L	↓	RAC2(K48) ERBB3 ULK1	ERK MAPK	RAC2/ERBB3 accumulation → MAPK ↑; ULK1 destabilization → excessive autophagy	(39, 40, 42)
	HERC1	↓	C-RAF (K48)	ERK	C-RAF stabilization → ERK ↑, proliferation ↑	(43)
	HERC2	↓	C-RAF MKK3	MKK3	Hyper-activation of C-RAF/MKK3/p38 axis, impaired oxidative stress response	(44)
	CHIP	↓	P21	AKT/p21	AKT phosphorylation↓, p21 expression↑	(91)
	TRIM21	↓	HIF-1α SREBF1 AXL(K48) ASS1(K63)	HIF-1α	Multi-axis degradation failure: glycolysis ↑, lipogenesis ↑, EMT ↑, immune suppression ↑	(60–63)
	TRIM44	↑	FRK Vimentin (K48)	FRK	Ubiquitin-mediated degradation of FRK/Vimentin → EMT suppression	(65, 66)
	UBR7	↓	–	–	Loss of tumor suppressor function → shorter overall survival	(93)
Oncogenic-OTU	OTUB1	↑	FOXM1 PD-L1(K48)	ECT2-Rho signaling	FOXM1-ECT2-Rho proliferation axis ↑ + PD-L1 immune evasion ↑	(113, 114)
	OTUD6A	↑	CDC6 (k6, 33, 48)	ATR-Chk1	CDC6 deubiquitination → DNA repair ↑, chemoresistance ↑	(116)
	OTUD6B	–	VHL	HIF-2α	OTUD6B ↓ → stabilization of mutated VHL →HIF-2α↑, migration↑	(118)
	OTUD3	↑	SLC7A11	Ferroptosis	SLC7A11 stabilization → Ferroptosis inhibition, ROS ↓, proliferation ↑	(115)
Tumor Suppressor-OTU	OTUD1	↓	PTEN STAT3	PTEN STAT3	OTUD1 ↓ → PTEN-STAT3 destabilization → PI3K/AKT ↑, PD-L1 ↑, immune suppression	(111, 112)
Oncogenic-UCH	UCHL5	↑	Snail1	B cells and antigen-representation	Snail1 deubiquitination → EMT ↑, impaired B cell antigen presentation	(123)
Tumor Suppressor-UCH	BAP1	Loss	HCF-1,chromatin remodeling complex	–	BAP1 inactivation → HCF-1 destabilization → cell cycle dysregulation	(121, 122)
Oncogenic-USP	USP7	↑	ARMC5 HIF-2α	HIF-2α	FUBPs-USP7-HIF-2α feed-forward loop → G1/S progression & tumor growth ↑	(99, 100)
	USP13	–	ZHX2	NF-κB	ZHX2-NF-κB target reshaping → Anchorage-independent growth ↑	(101)
	USP22	↑	Survivin	Apoptosis	Survivin deubiquitination → Apoptosis inhibition	(106)
	USP35	↑	IAPs NRF2	Ferroptosis	NRF2-IAP stabilization → Ferroptosis evasion + glycerophospholipid reprogramming	(102, 103)
	USP37	–	HIF-2α	HIF-2α	USP37–HIF-2α axis → Proliferation + spontaneous lung metastasis	(107)
	USP39	↑	AKT-pS473SRPK1	AKT	Sustained AKT activation + VEGF-A165b splicing shift → Angiogenesis & migration ↑	(104, 105)
	USP10	↓	p53	P53	p53 destabilization → growth de-repression	(108)
Tumor Suppressor-USP	USP44	↓	p21 Cyclin D1 JNK	JNK	p21 destabilization + JNK hyper-activation → cycle dyscontrol	(109)
	USP53	↓	IκB	NF-κB	Loss of NF-κB restraint → proliferation & metastasis ↑	(110)

The symbol “↑↓” denotes an increase and a decrease.

**Figure 3:**
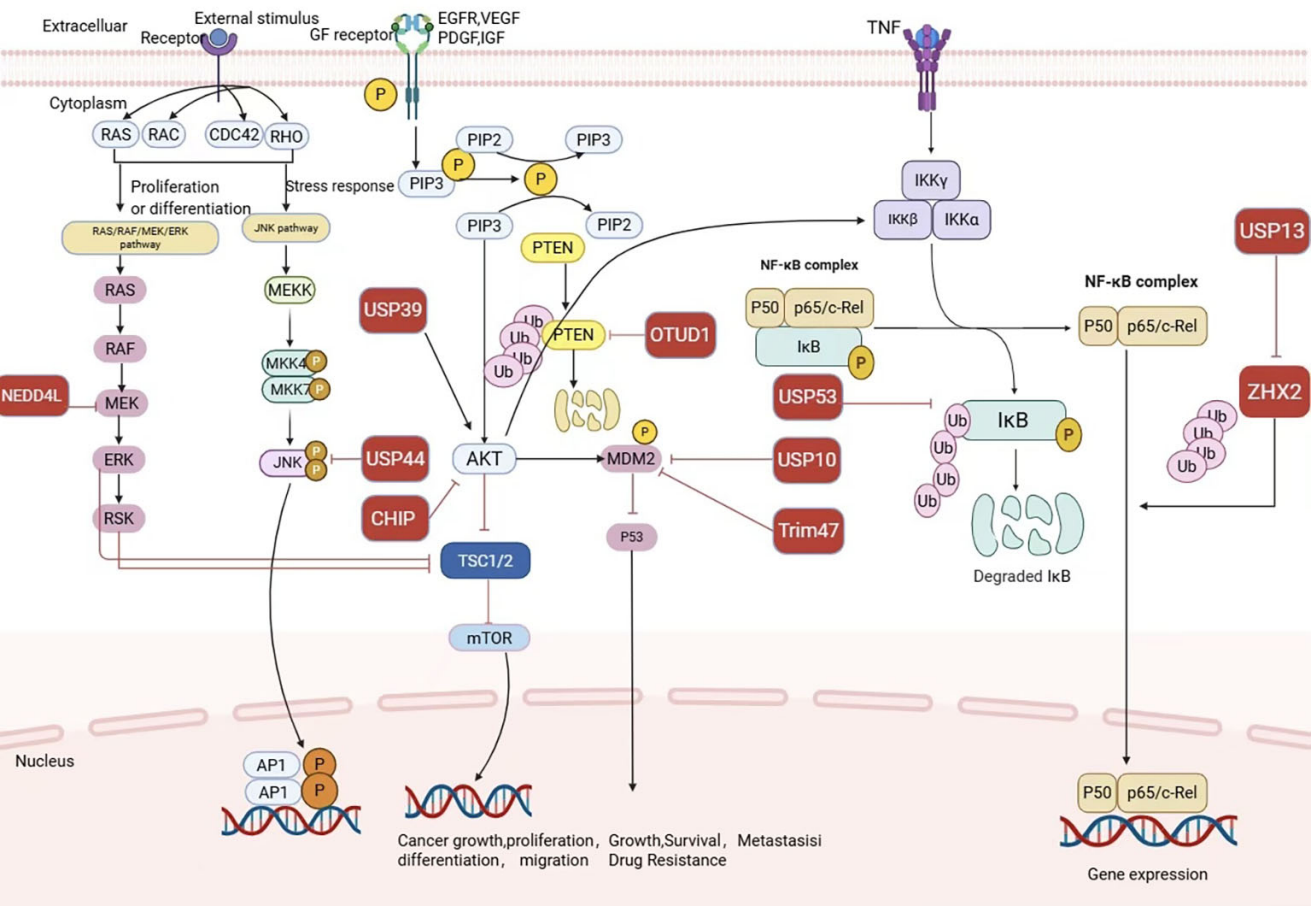
Core Signaling Pathways of E3 Ligases and Deubiquitinases in Renal Cell CarcinomaDiagram showing cell signaling pathways involved in proliferation,differentiation, stress response, and NF-kB complex. Includes RAS/RAF/MEK/ERK, JNK,PI3K/AKT/mTOR pathways, and ubiquitination processes. Highlights proteins like MEKK,AKT, PTEN, IKKa, IKKb, and downstream effects such as cancer growth, gene expression, metastasis, and drug resistance. Various proteins, receptors, and enzymes are depicted with interactions and phosphorylations annotated.

The roles of these E3 ligases and deubiquitinases in RCC are summarized in **Table 2**, and the core signaling pathways they are involved in are shown in (**Figure 3**).

## 5 Therapeutic strategies targeting key ubiquitination enzymes in RCC

For VHL-deficient ccRCC, anti-VEGF/TKI therapy remains the first-line standard. Bevacizumab combined with IFN-α, as well as sunitinib, axitinib, and other agents, blocks the HIF-downstream VEGF/PDGF axis, significantly extending median progression-free survival (PFS) to 11–13 months (123, 124). However, most patients develop resistance within 12–18 months, with second-line options limited to the mTOR inhibitor everolimus or cross-line TKI continuation.

In recent years, HIF-2α inhibitors have provided breakthroughs for later-line therapy in patients with TKI/mTOR inhibitor failure or VHL syndrome. The HIF-2α inhibitor belzutifan (PT2977) offers a novel alternative. The Phase III LITESPARK-005 trial demonstrated that belzutifan significantly prolonged median PFS versus everolimus (14.6 vs 7.2 months, HR 0.62) and achieved an ORR of 49% (125). Consequently, the ccRCC treatment paradigm is shifting from single-agent VEGF targeting to sequential/combined VEGF→HIF-2α strategies. Future research must explore optimal windows and biomarkers for belzutifan-TKI/immunotherapy combinations.

However, the long-term efficacy of existing targeted therapies remains limited by resistance mechanisms. In recent years, the pivotal role of the ubiquitin-proteasome system in regulating tumor immune responses has become increasingly prominent, providing a new perspective for understanding and overcoming immunotherapy resistance in RCC. For instance, OTUB1 is highly expressed in RCC tissues and positively correlates with PD-L1 abundance; it blocks ERAD-mediated degradation through deubiquitinating PD-L1 K48 chains, thereby enhancing PD-1 binding and inhibiting CD8^+^ T cell infiltration and IFN-γ secretion (114). NFAT1 is stabilized through hyperactivation of PI3K/AKT/GSK-3β, upregulates PD-L1, promotes immune evasion, and mediates sunitinib resistance (52). These findings collectively reveal that the ubiquitin system serves as a central hub connecting RCC biology with tumor immunity, suggesting that targeting specific E3 ligases or deubiquitinases represents a highly promising strategy for combination immunotherapy. However, no relevant inhibitors have entered clinical trials yet.

In terms of innovative strategies for immunotherapy, the novel antigen-targeted degradation tumor vaccine (TAgD-TVac) employs lymph node-targeting lipid nanoparticles conjugated with E3 ligands and antigens, leveraging the ubiquitin-proteasome pathway to enhance antigen processing and cross-presentation. Preclinical models confirm that TAgD-TVac induces specific adaptive immunity and immunological memory, effectively inhibiting tumor growth, metastasis, and recurrence while synergizing with immune checkpoint blockade therapy (126).

Thus, current strategies are evolving from sole VEGF targeting toward sequential/combined HIF-2α inhibitors, mTOR inhibitors, and immune microenvironment modulation, while innovative E3/DUB-targeted tumor vaccines provide new avenues to overcome resistance. Future studies must further dissect substrate selectivity and spatiotemporal specificity of ubiquitination modifications and identify optimal windows and biomarkers for combinatorial interventions.

## 6 Summary and perspectives

This review primarily explores how ubiquitination, through E3 ligases and deubiquitinating enzymes (DUBs), regulates protein stability and signal transduction, playing critical roles in RCC with significant clinical implications. We systematically summarized the classification, structural features, and functional roles of E3 ligases and DUBs in RCC. Studies demonstrate that different E3 subfamilies (e.g., HECT, RING, U-box, PHD) and DUBs (e.g., USP, OTU, UCH) exhibit an imbalanced pattern in RCC, where oncogenic members are upregulated while tumor-suppressive members are downregulated, extensively participating in critical processes including cell proliferation, apoptosis, autophagy, metabolic reprogramming, immune evasion, and metastasis. For instance, Smurf2, MDM2, and USP7 promote tumor progression by stabilizing oncoproteins or degrading tumor suppressors, whereas FBXW7, TRIM21, and USP10 exert protective effects by negatively regulating oncogenes or stabilizing tumor suppressive factors. Additionally, VHL, as the most representative E3 ligase in ccRCC, profoundly influences tumor metabolism, immune microenvironment, and therapeutic response through HIF pathway activation upon following inactivating mutations, representing a critical therapeutic target. These studies highlight the therapeutic potential and promise of ubiquitination-related enzymes in RCC treatment. Despite significant advances in E3 ligase and DUB research in RCC, numerous challenges remain. For example, the substrate repertoire of most E3s/DUBs remains incompletely characterized, and the mechanisms underlying ubiquitin chain type specificity (K48, K63, etc.), spatiotemporal regulation, and their relationship with tumor heterogeneity require further investigation. Future research should focus on the following two-pronged approach: At the mechanistic level, continued investigation into the specific molecular mechanisms underlying dysregulation of ubiquitination-related enzymes; integration of ubiquitinomics, single-cell sequencing, and CRISPR screening technologies to generate a panoramic E3/DUB-substrate interaction atlas in RCC; and clarification of the spatiotemporal functional heterogeneity of E3/DUBs across different RCC subtypes, tumor microenvironment crosstalk, and the evolution of therapeutic resistance. These studies will deepen understanding of RCC pathogenesis and provide novel targets for precision therapy. At the clinical level: accelerated development of small-molecule inhibitors or specific degraders targeting E3/DUBs (such as PROTACs and DUBTACs), with exploration of combination strategies with immune checkpoint inhibitors, targeted agents, or metabolic modulators; evaluation of the clinical value of ubiquitination-related biomarkers (e.g., VHL mutation status, specific DUB expression levels) for patient stratification, prognostic prediction, and therapeutic monitoring; and development of individualized treatment regimens targeting core pathways such as VHL-HIF. As translational research advances, targeting the ubiquitin system is poised to become a pivotal breakthrough in the precision diagnosis and treatment of RCC.
